# Minutes of the Cincinnati Local Dental Association

**Published:** 1860-12

**Authors:** 


### Proceedings of Societies.

### MINUTES OF THE CINCINNATI LOCAL DENTAL ASSOCIATION.

CINCINNATI, Tuesday Evening, Nov. 13, 1860.

Local Dental Association met pursuant to adjournment at the office of Dr. Cameron.

*Members present*—Drs. Richardson, Taft, H. R. Smith, H. A. Smith, Wells, Wheeler, Cameron and Davenport.

Minutes of the previous meeting read and approved.

On motion of Dr. RICHARDSON, the rules were suspended to take some action in reference to the death of Prof. CHAPIN A. HARRIS, of Baltimore.

On motion of Dr. Richardson, a committee of three was appointed to draft resolutions expressive of the feelings of the members of this Association, in reference to the above. Drs. Richardson, Taft and H. R. Smith being appointed retired, and shortly presented the following preamble and resolutions, which were adopted, viz:

*Whereas*, The members of this Association have heard with emotions of profound sorrow of the death of Prof. Chapin A. Harris, of Baltimore, it is most proper, that as members of the same profession, we should give fitting expressions to the sentiments of grief which effect all hearts alike in this great calamity; and,

*Whereas*, In obedience to a common impulse of love and gratitude, we desire earnestly to bear testimony to the eminent rectitude of character which distinguished the private and professional relations of Dr. Harris in life, and to express our appreciation of the great and distinguished services which have deservedly won for him the proud title of "Father of American Dentistry," therefore,

*Resolved*, That we deplore the event which has filled the hearts of his bereaved family with a sorrow inconsolable, and which has robbed the profession of one of its best, most able and venerated professors, and mankind of one of its leading and most distinguished benefactors.

*Resolved*, That in the private life and character of Dr. Harris are exemplified those virtues that command our unqualified respect and admiration, and are in all respects worthy of studied imitation.

*Resolved*, That the great and untold blessings and benefits, which, in the several capacities of author, teacher, and practitioner, Dr. Harris has conferred on mankind and the profession through thirty years of laborious and unremitting devotion to the cause of Dental Science, merits and receives our warmest gratitude, and for which his honored name shall remain forever "green in our memories."

*Resolved*, That we tender to the family of the deceased our heartfelt sympathies in their great affliction, and the Secretary be instructed to forward to the same a copy of these resolutions.

*Resolved*, That we extend to Prof. James Taylor an invitation to prepare and deliver to this Association, at its next regular meeting, an address upon the life and character of the deceased.

J. Richardson,

J. Taft,

H. R. Smith.

Dr. Richardson was appointed to wait upon Dr. Taylor and inform him of the wish of the Association, as above.

The discussion of the question, "How soon after the extraction of the natural teeth should artificial dentures be inserted?" was then opened by Dr. Richardson, who read a paper upon the subject. The paper being incomplete he was requested to complete and hand it to the Publishing Committee.

Dr. Taft was appointed to act on the committee in the room of Dr. Richardson.

Dr. Richardson referred, *verbally*, to an objection sometimes urged against the very early insertion of artificial dentures, that "the plate would prove an irritation to the gums." He thought this was a mistake, that the plate would be a protection rather than otherwise.

Dr. H. R. Smith, considers the circumstances of each case. In partial plates prefers to wait, because if it be an atmospheric pressure plate, the change in the gums will soon loosen the plate; if cheap work, the cheap teeth will soon have to sustain all the weight of the plate. In full dentures recommends immediate insertion, owing to the action of the masseter and buccinnator muscles, the proper angle of the jaws is destroyed in the patient who goes without teeth. After extracting the natural teeth, trims off the alveolus rather than the gum. In making substitutes fills up the sockets on the model. Refits the plate after two or three months. Unless this can be done, dissuades from temporary work. Have refitted a plate as often as three times. Thinks the time can not be fixed when the gums have entirely ceased changing. Unless temporary work can be inserted immediately, prefers to wait two weeks.

Dr. Wheeler sometimes inserts teeth immediately, prefers to wait two or three weeks. The relation of the jaws and the expression of the face is better preserved by temporary work. Does not allow the plate to extend into the sockets of the natural teeth.

Dr. Wells inquired if there was not danger in trimming the alveolus, of producing too much absorption.

Dr. H. R. Smith thinks not. The object is to trim no more than would be removed by the process of absorption.

Dr. Richardson—After extraction,patients often complain of pain around the sockets of the cuspid teeth, which may be relieved by dividing the mucous membrane, and breaking down the process. Does not think cutting away of the process will make any difference in the form of the ridge, unless the cutting has been too deep.

Dr. Cameron—Wait from ten days to two weeks—never allow the plate to extend over the outside of the gum. Generally use gum teeth.

Dr. Taft inserts as soon as possible. The changes in the use and motion of the lower jaw begin at once and should be remedied. Persons without teeth soon lose the proper use of the lower jaw. There is less soreness of the gums if the teeth are inserted at once, than if otherwise. The early insertion relieves pronunciation. Sometimes use gum and at others plain teeth. Mentioned a case of a lady, seventy-five years of age, for whom he inserted a full set of continuous gum work, two or three weeks after extraction. In a short time she spoke very distinctly. They would often rattle in the mouth during mastication—substituted rubber in the upper jaw—difficulty not removed—is unable to account for it.

Dr. H. R. SMITH referred to a similar case.

Dr. TAFT thinks temporary partial pieces are not as necessary as full pieces, but generally inserts them.

Dr. RICHARDSON—In twenty-four or forty-eight hours after extraction, the gums become sore and tumefied. Thinks a plate inserted then would give pain.

Dr. TAFT—There is not, *generally*, any tumefaction, unless in an inflammatory diathesis; even if there is, the pain caused by the insertion of a plate, does not last longer than two or three minutes.

Dr. H. A. SMITH—Practice very similar to the others. Thinks the upper teeth are retained with more difficulty if not immediately inserted. Finds trouble also in obtaining a correct bite. Does not think the plate being swaged into the socket will hinder the deposition of matter.

Dr. TAFT suggested that lining the plate with rubber was better than re-swaging; gives a good fit and saves re-arranging the teeth.

Some cases of *so-called* painless extraction of teeth were given by different members.

Dr. H. A. SMITH presented a case of irregularity, and asked the best method of treatment. Opinions were given by different members.

Dr. MERIT WELLS was appointed essayist for next meeting.

Question for discussion—"What are the conditions indicating the extraction of the deciduous teeth, and the best method of performing the operation.

Adjourned to meet at the Dental College, on the 2d Tuesday evening of December. T. F. DAVENPORT, *Sec'y.*

